# Leveraging Large Language Models and Machine Learning for Success Analysis in Robust Cancer Crowdfunding Predictions: Quantitative Study

**DOI:** 10.2196/73448

**Published:** 2025-11-19

**Authors:** Runa Bhaumik, Abhishikta Roy, Vineet Srivastava, Lokesh Boggavarapu, Ranganathan Chandrasekaran, Edward K Mensah, John Galvin

**Affiliations:** 1 Department of Psychiatry, College of Medicine, University of Illinois Chicago, 1601 West Taylor Street, Chicago, IL, 60612, United States, 1 7085672467; 2 Department of Information and Decision Sciences, University of Illinois Chicago, Chicago, IL, United States; 3 Health Policy and Administration, School of Public Health, University of Illinois Chicago, Chicago, IL, United States; 4 Division of Hematology and Oncology, Department of Medicine, College of Medicine, University of Illinois Chicago, Chicago, IL, United States

**Keywords:** cancer crowdfunding, machine learning, large language models, linguistic features, social determinants of health, health policy

## Abstract

**Background:**

Recent advances in large language models (LLMs) such as GPT-4o offer a transformative opportunity to extract nuanced linguistic, emotional, and social features from medical crowdfunding campaign texts at scale. These models enable a deeper understanding of the factors influencing campaign success far beyond what structured data alone can reveal. Given these advancements, there is a pressing need for an integrated modeling framework that leverages both LLM-derived features and machine learning algorithms to more accurately predict and explain success in medical crowdfunding.

**Objective:**

This study addressed the gap of failure to capture the deeper psychosocial and clinical nuances that influence campaign success. It leveraged cutting-edge machine learning techniques alongside state-of-the-art LLMs such as GPT-4o to automatically generate and extract nuanced linguistic, social, and clinical features from campaign narratives. By combining these features with ensemble learning approaches, the proposed methodology offers a novel and more comprehensive strategy for understanding and predicting crowdfunding success in the medical domain.

**Methods:**

We used GPT-4o to extract linguistic and social determinants of health features from cancer crowdfunding campaign narratives. A random forest model with permutation importance was applied to rank features based on their contribution to predicting campaign success. Four machine learning algorithms—random forest, gradient boosting, logistic regression, and elastic net—were evaluated using stratified 10-fold cross-validation, with performance measured through accuracy, sensitivity, and specificity.

**Results:**

Gradient boosting consistently outperformed the other algorithms in terms of sensitivity (consistently 0.786 to 0.798), indicating its superior ability to identify successful crowdfunding campaigns using linguistic and social determinants of health features. The permutation importance score revealed that for severe medical conditions, income loss, chemotherapy treatment, clear and effective communication, cognitive understanding, family involvement, empathy, and social behaviors play an important role in the success of campaigns.

**Conclusions:**

This study demonstrates that LLMs such as GPT-4o can effectively extract nuanced linguistic and social features from crowdfunding narratives, offering deeper insights than traditional methods. These features, when combined with machine learning, significantly improve the identification of key predictors of campaign success, such as medical severity, financial hardship, and empathetic communication. Our findings underscore the potential of LLMs to enhance predictive modeling in health-related crowdfunding and support more targeted policy and communication strategies to reduce financial vulnerability among patients with cancer.

## Introduction

The cost of cancer care in the United States is increasing rapidly due to various factors, including technological advancements, expensive cutting-edge therapies, and improved access to treatment. Patients with cancer and their families often face substantial financial consequences, such as borrowing money, spending less on food, going into debt, or declaring bankruptcy [1-3]. These issues are exacerbated by a lack of medical insurance or lack of coverage due to health care regulations such as the Affordable Care Act. As a result, patients and caregivers seek help from other sources to raise funds for medical care [2].

Crowdfunding has become a significant tool for raising money through social media and web-based platforms such as Indiegogo, Kickstarter, and GoFundMe, generating US $34.4 billion in 2015 alone [4-7]. Medical crowdfunding constitutes a significant portion of these campaigns in the United States due to gaps in insurance coverage and the prohibitive costs of medical treatments, such as in cancer care, even for those with insurance [8,9]. Success factors for these campaigns include demographic attributes, racial background, campaign images, textual features, and social media characteristics, with campaigns for children attracting more donations than those for adults [6,7,9,10]. In cancer treatment crowdfunding, 65.4% of campaigns involve advanced-stage patients, with detailed information about cancer types, treatments, and costs leading to higher fundraising success [11-15]. In addition, research has characterized the use of crowdfunding to support oncology care needs, examining associations between insurance status and other characteristics related to unmet financial obligations [13].

The field of crowdfunding prediction has primarily relied on traditional statistical methods such as linear and logistic regression in previous studies. These methods assume that the input variables are independent from each other. However, when there are correlations among the input variables, using regression methods can lead to larger prediction errors. To overcome this limitation, researchers have turned to machine learning (ML) techniques. ML algorithms have proven to be effective in analyzing hidden associations within large datasets and identifying complex patterns [16,17]. As a result, researchers have used ML algorithms to predict the success of crowdfunding campaigns [18-20]. This shift in methodology allows for better prediction accuracy and the discovery of more nuanced relationships among the input variables. For instance, ML algorithms such as support vector machine, decision tree, and k-nearest neighbor have been used to predict the success of projects [18,19,21-24]. In addition, algorithms such as extreme gradient boosting, gradient boosting, random forest, and generalized linear models have been used to construct prediction models [25].

Previous crowdfunding research has demonstrated that ML methods can effectively model complex relationships between variables, capture interactions, and yield more accurate and insightful predictions. However, there has been a lack of thorough analysis regarding the selection of predictors and campaign success measures. Furthermore, the application of large language models (LLMs) in the crowdfunding domain remains relatively unexplored. The only study in this domain to date [26] has generated insights on medical financial hardship patterns and unmet social needs by leveraging generative artificial intelligence on GoFundMe cancer crowdfunding campaigns using logistic regression.

In this study, we explored the use of LLMs and ML models to generate and identify predictors of successful campaigns. LLMs are particularly helpful in capturing the semantic meaning of words and phrases in internet-based texts by using word embeddings and contextual embeddings, which were trained on large, diverse texts. GPT-4o [27], developed by OpenAI, is an LLM known for its conversational text generation capabilities. We used GPT-4o and prompted relevant campaign information to extract and analyze linguistic and social determinants of health (SDOH) factors. We also provided a robust feature selection based on the permutation technique for a better understanding of the model.

Our research had three primary objectives: (1) to leverage LLMs, specifically GPT-4o, to automatically generate a wide range of linguistic, social, and clinical features from crowdfunding campaign narratives; (2) to implement a robust feature selection strategy using the random forest algorithm combined with permutation importance to quantify and interpret the relative contribution of each feature to predictive performance without implying causal relationships; and (3) to conduct a comprehensive evaluation of predictive performance across multiple ML algorithms using the key features identified through the random forest–based feature selection approach.

To the best of our knowledge, this study is the first to leverage an LLM for feature extraction and an ML algorithm to establish an effective prediction model for GoFundMe cancer crowdfunding campaigns with a robust feature selection method. By integrating innovative ML algorithms and feature selection strategies into crowdfunding research, we not only advance the academic understanding of this domain but also provide actionable insights that can be directly applied by policymakers and campaign organizers. These advancements are essential for addressing the growing reliance on crowdfunding in the health care sector and ensuring that support reaches those who need it the most.

## Methods

### Ethical Considerations

All data used in this study were obtained from publicly accessible crowdfunding campaigns on the GoFundMe platform via its public API. The API provides campaign metadata (eg, title, goal amount, amount raised, description text, and campaign category) that are already visible to any online user without registration. No private, donor-level, or personally identifiable information (PII) was accessed, and no authentication was required. The research complied with GoFundMe’s published Terms of Service [28] at the time of data collection. Because this study only used publicly accessible data, institutional review was not required, as per local guidelines [29].

### Data Collection

The data for this study were collected from the GoFundMe crowdfunding platform, which is the world’s largest platform in terms of both the total amount of funding raised and the total number of active campaigns. Founded in 2010, GoFundMe features campaigns for various categories, including medical, memorials, emergencies, and charitable causes. The data for this analysis were extracted using a combination of application programming interface (API) access and web scraping techniques. GoFundMe stores its data in Algolia, a search and analytics engine. We obtained the necessary API key, secret key, and index name by inspecting the network tab on the GoFundMe website. We connected to the Algolia API using a Python (Python Software Foundation) program and retrieved the data. The API provided access to all records stored in the specified index, which we then processed and stored for further analysis. The data consist of 4990 campaigns for patients’ oncologic treatment and personal and financial lives that began between January 3, 2023, and December 31, 2023, with funding targets between US $800 and US $100,000.

### Campaign Success Measure

We considered a campaign to be successful if it raised a significant percentage of the goal amount. The success ratio was defined as the ratio of funds raised to the campaign goal and was converted to a binary variable at different success thresholds. In this experiment, we selected the success threshold as a ratio of 0.7 based on the observations in exploratory analysis.

### Predictors of Campaign Success

#### Crawled Variables

We collected key campaign data, including launch date, title, description, current raised amount, target goal, number of donors, and campaign duration. These details provide insights into each campaign’s progress and engagement.

#### SDOH and Medical-Related Variables

The selected variables were obtained from the Linguistic Inquiry and Word Count (Pennebaker Conglomerates, Inc) internal dictionary [30] and the Centers for Disease Control and Prevention indicators [31]. In addition, we incorporated several new variables related to medical procedures and treatment factors to enhance the dataset. A full categorization of these variables is provided in Table 1.

**Table 1.** Categorization of the extracted features used for crowdfunding campaign analysis.

Category	Factors
Cognitive and psychological	Analytic thinking, cognition, tone, risk, reward, empathy, politeness, and authenticity
Linguistic and communication	Clout, linguistic content, communication, present tense, past tense, and future tense
Medical and health	Cancer site, stage or grade of cancer, type of therapy, treatment status, treatment duration, engagement in health care activities, comorbidities, association with health care organizations and facilities (hospices, hospitals, nursing homes, skilled nursing facilities, and emergency rooms)
Emotional states	Stress, anxiety, mental or behavioral dysfunctions, chronic diseases, cognitive content of communications, expression of social concerns, informal language (swear words and online abbreviations), sentiment (positive, negative, or neutral), and trust in medical care facilities
Employment and education	Employment status, work disruption (for individuals aged ≥18 years), school absenteeism, work absenteeism, and parents’ work disruption (for beneficiaries aged <18 years)
Financial and medical support	Lack of sick leave, lack of medical attention, income loss, family income loss, struggles with medical expenses, housing expenses, food expenses, and transportation expenses
Medical procedures and treatments	Laboratory procedures, diagnostic procedures, cancer-specific treatments (chemotherapy and radiotherapy), preventive procedures, alternative physical therapy, occupational therapy, and mental health or substance abuse therapy
Organizations and facilities	Association with health care–related organizations, professional societies, self-help organizations, relief organizations, and receiving care in specific facilities (hospices, hospitals, nursing homes, skilled nursing facilities, emergency rooms, or mental health or substance abuse facilities)
Mental state, sentiment, or trust	Presence of stress, anxiety, mental or behavioral dysfunctions, or chronic diseases; sentiment (positive, negative, or neutral); and level of trust in medical care facilities

### Experimental Setup

#### Feature Extraction and Selection Methodology

The variables were programmatically generated using GPT-4o, a state-of-the-art LLM from OpenAI, through a structured prompting pipeline designed to extract nuanced semantic, emotional, and contextual signals and other factors from campaign narratives. We used a zero-shot prompting strategy tailored to identify all the variables. For example, the following was a representative prompt used: “Analyze the following crowdfunding campaign description and return the sadness (0 -low and 1 -high) score or whether patient struggles with expenses for medical treatments (yes or no),).”

We manually evaluated GPT-4o–based feature outputs for a random sample of 50 crowdfunding campaigns to assess consistency and accuracy. Each campaign was processed using the GPT-4o API with temperature set to 0.5, maximum tokens limited to 256, and top_p (probabilistic decoding parameter) set to 1.0 to ensure stable and contextually appropriate responses. Manual review involved comparing GPT-4o–extracted linguistic and SDOH features against human-annotated references. The observed validation error ranged from 0.06 to 0.08, indicating high reliability and supporting the use of GPT-4o for automated feature generation in this study.

Postprocessing routines were developed to convert the generated responses into structured binary or ordinal variables, which were then added to the campaign-level dataset. These extracted features were then categorized, standardized, and incorporated into downstream statistical and ML models to quantify their relative importance. Importantly, we separately evaluated the contributions of linguistic and SDOH feature sets using permutation-based feature importance within an ensemble random forest classifier, ensuring that observed effects were interpreted as associations within predictive contexts and not causal mechanisms.

We applied minimum-maximum normalization to variables with large value ranges (target amount, raised amount, number of donors, and campaign duration) to minimize their impact on prediction results. GPT-4o was used to standardize measures of linguistics and campaign predictors between 0 and 1. The predictors for social and demographic identity, as well as medical-related predictors, were extracted as a binary (yes or no) and dummy coded.

#### ML Algorithms

We conducted 2 comparative ML experiments to evaluate the predictive utility of different feature sets in determining the success of cancer-related crowdfunding campaigns. In both experiments, the dataset was divided into training (70%) and testing (30%) subsets, and all models were validated using 10-fold stratified cross-validation on the training data to ensure robustness and minimize overfitting.

In the first experiment, we focused on linguistic and campaign-level features. Linguistic features—such as tone, empathy, clout, and sentiment—were extracted using GPT-4o through structured prompts. These were combined with campaign-level metadata such as fundraising goals, campaign duration, and update frequency. To evaluate the contribution of individual features to prediction performance, we initially trained a random forest classifier using the scikit-learn library (Google Summer of Code project) on the training dataset, with the number of trees set to 100 and the minimum samples required to split an internal node (min_samples_split) set to 2. These parameter values were selected based on a grid search optimization process in which they achieved the highest accuracy (0.72). For feature importance, we used permutation importance, a model-agnostic approach that measures the decline in model performance when each feature is randomly shuffled. This method was applied within the training set to assess the relative contribution of each feature. The features were then ranked by their permutation importance scores. On the basis of these rankings, we constructed a series of feature subsets including the top 3, 6, 9, 12, and 15 features.

In the second experiment, we applied the same methodology to a different set of features—those reflecting social, demographic, and medical information. These included SDOH (eg, references to employment status, caregiving responsibilities, and financial hardship), demographic attributes (eg, inferred race or ethnicity), and medically relevant content (eg, mentions of chemotherapy, hospice care, or immunotherapy). These features were similarly extracted using GPT-4o with custom prompts designed to infer relevant categories from the narrative descriptions in the campaign text. As with the first experiment, random forest with permutation-based feature importance was used to rank features within the training set, and model performance was assessed at various thresholds of top N features.

For both experiments, we also evaluated the performance of 3 additional ML models: gradient boosting (min_samples_split=2; n_estimators=100), logistic regression, and elastic net (solver=“saga”; l1_ratio=0.5) with default parameter settings. We evaluated model performance using subsets of the highest-ranked features, testing configurations with 3, 6, 9, 12, and 15 features to provide a comparative perspective. Model performance was assessed using 3 evaluation metrics: accuracy, sensitivity, and specificity. Sensitivity was defined as the ratio of true positives (successful campaigns) to the sum of true positives and false negatives, specificity was defined as the ratio of true negatives (unsuccessful campaigns) to the sum of true negatives and false positives, and accuracy was defined as the proportion of correct predictions out of all predictions made.

## Results

### Descriptive Analysis

In our analysis, we used a dataset of crowdfunding campaigns to explore their success in relation to their progress toward the established goal amounts. The descriptive statistics of the raised amounts provided valuable insights into the overall distribution and characteristics of the dataset. Among the 4990 campaigns analyzed, the mean amount raised was US $20,016.67 (SD US $26,456.97), with a median of US $11,819 (IQR US $17,383). Similarly, the mean goal amount among the scraped links was US $40,373.73 (SD US $55,043.88), and the median was US $25,000 (IQR US $38,000). We divided the dataset into 5 distinct groups by categorizing the data into percentile ranges based on the distribution of goal amounts. Each group was of equal size: US $799.99 to US $10,000, US $10,000 to US $20,000, US $20,000 to US $30,000, US $30,000 to US $50,000, and US $50,000 to US $100,000.

**Figure 1.**
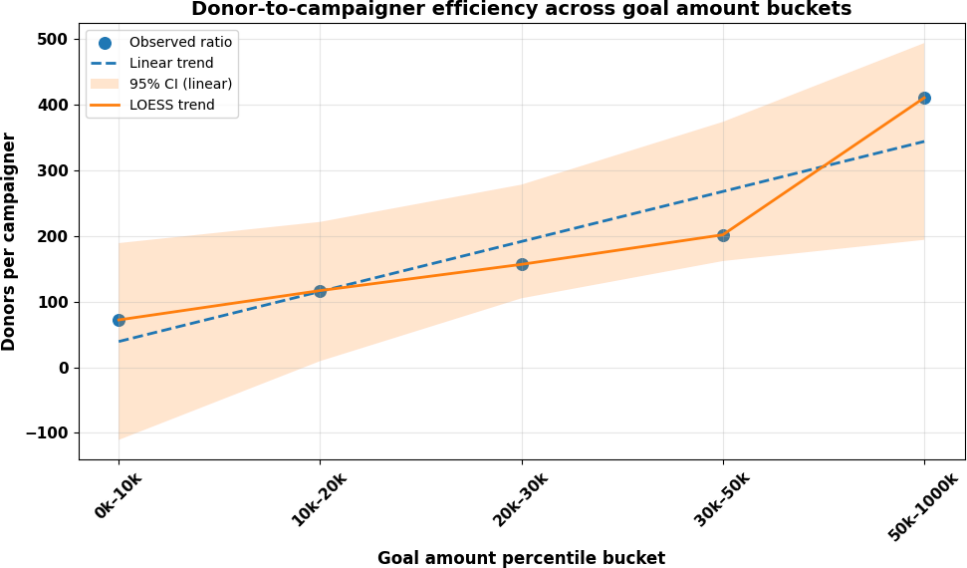
Donor-to-campaigner efficiency curve for medical crowdfunding campaigns. Linear trend: slope=304.167; intercept=35.000; *R*^2^=0.966; *P*=.003. LOESS: locally estimated scatterplot smoothing.

Figure 1 illustrates that the donor-to-campaigner ratio increased steadily across goal-amount percentiles, indicating that campaigns with larger targets typically mobilized more donors on average. The alignment between the linear and locally estimated scatterplot smoothing curves suggests that this relationship is approximately linear, though a mild upward curvature at the upper end (80k-100k) may reflect heightened engagement for high-visibility or urgent medical cases. The broader confidence band among large-goal campaigns implies heterogeneity in donor response, consistent with the notion that some high-goal efforts achieve viral reach while others fail to gain traction. Overall, this pattern supports the hypothesis that higher fundraising targets often signaling greater medical severity or broader social networks are associated with enhanced donor participation efficiency.

**Figure 2.**
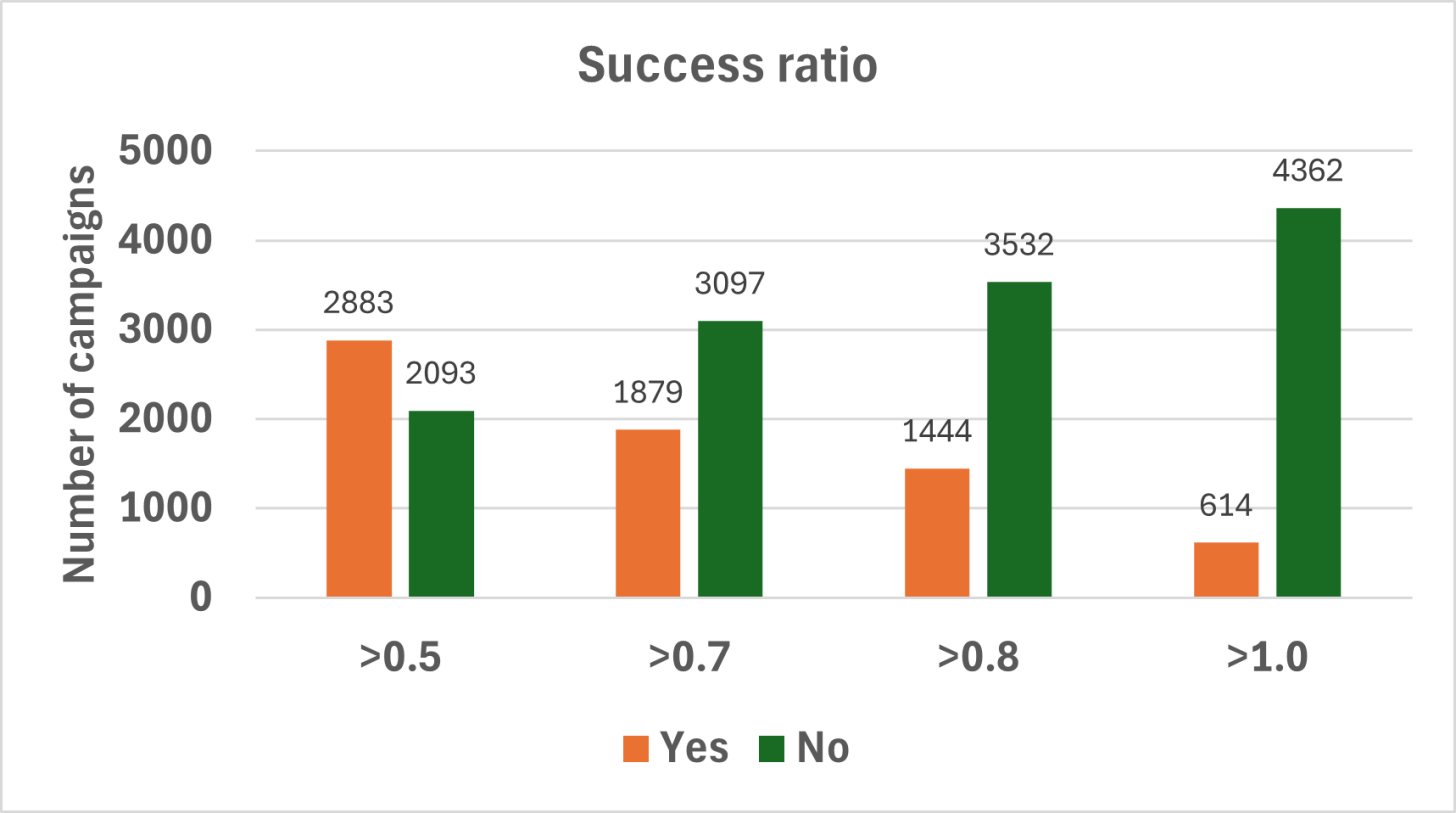
Distribution of crowdfunding campaigns based on their success ratio.

Figure 2 shows that as the success ratio threshold increases, the proportion of successful campaigns decreases, whereas the number of unsuccessful campaigns increases. This trend is expected as higher thresholds represent more ambitious funding goals, which are harder to achieve.

**Figure 3.**
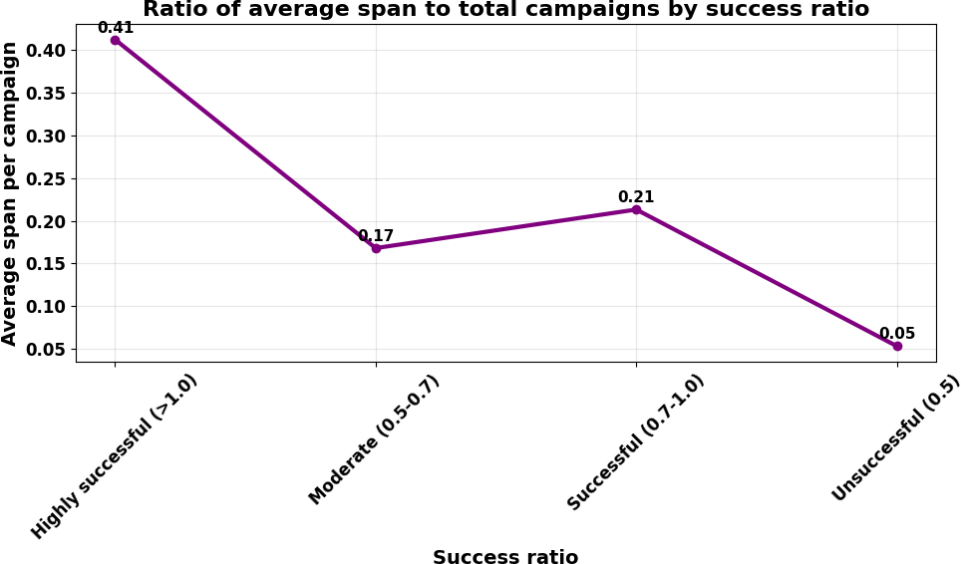
Ratio of average span to total campaigns by success ratio.

Figure 3 shows the ratio of mean campaign span (days) to total campaigns within each success-ratio category: unsuccessful (<0.5), moderate (0.5-0.7), successful (0.7-1.0), and highly successful (> 1.0). Campaigns achieving higher success ratios exhibited greater relative span durations (approximately 0.41 for highly successful vs approximately 0.05 for unsuccessful campaigns). The upward pattern indicates that prolonged visibility and sustained engagement are associated with improved crowdfunding outcomes, whereas unsuccessful campaigns tend to terminate earlier and attract fewer donors.

### Socioeconomic Variables and Their Association With Campaign Success

To assess the role of socioeconomic context in campaign outcomes, we analyzed 5 key variables derived from campaign narratives: work absenteeism, educational activity, friend, struggles with monthly bills, and struggles with food. Work absenteeism captured whether the individual referenced missing work due to illness or treatment, indicating employment disruption and potential income loss. Educational activity included references to school attendance or disruptions to education, suggesting that the campaign involved students or caregivers with educational responsibilities. The friend variable identified campaigns organized by or for a friend, providing insights into the patient’s social network and potential campaign reach. Struggles with monthly bills flagged mentions of difficulty affording recurring monthly expenses (eg, rent and utilities), serving as a direct marker of financial strain. Struggling with food indicated food insecurity, reflecting the inability to afford sufficient or nutritious food. Each of these variables was statistically tested for its association with campaign success using chi-square analysis. Although none reached statistical significance at *P*<.05, work absenteeism showed a marginal association (*χ*^2^_1_=4.7; *P*=.10), suggesting a potential trend worth further investigation.

### Campaign Success Determinants

**Figure 4.**
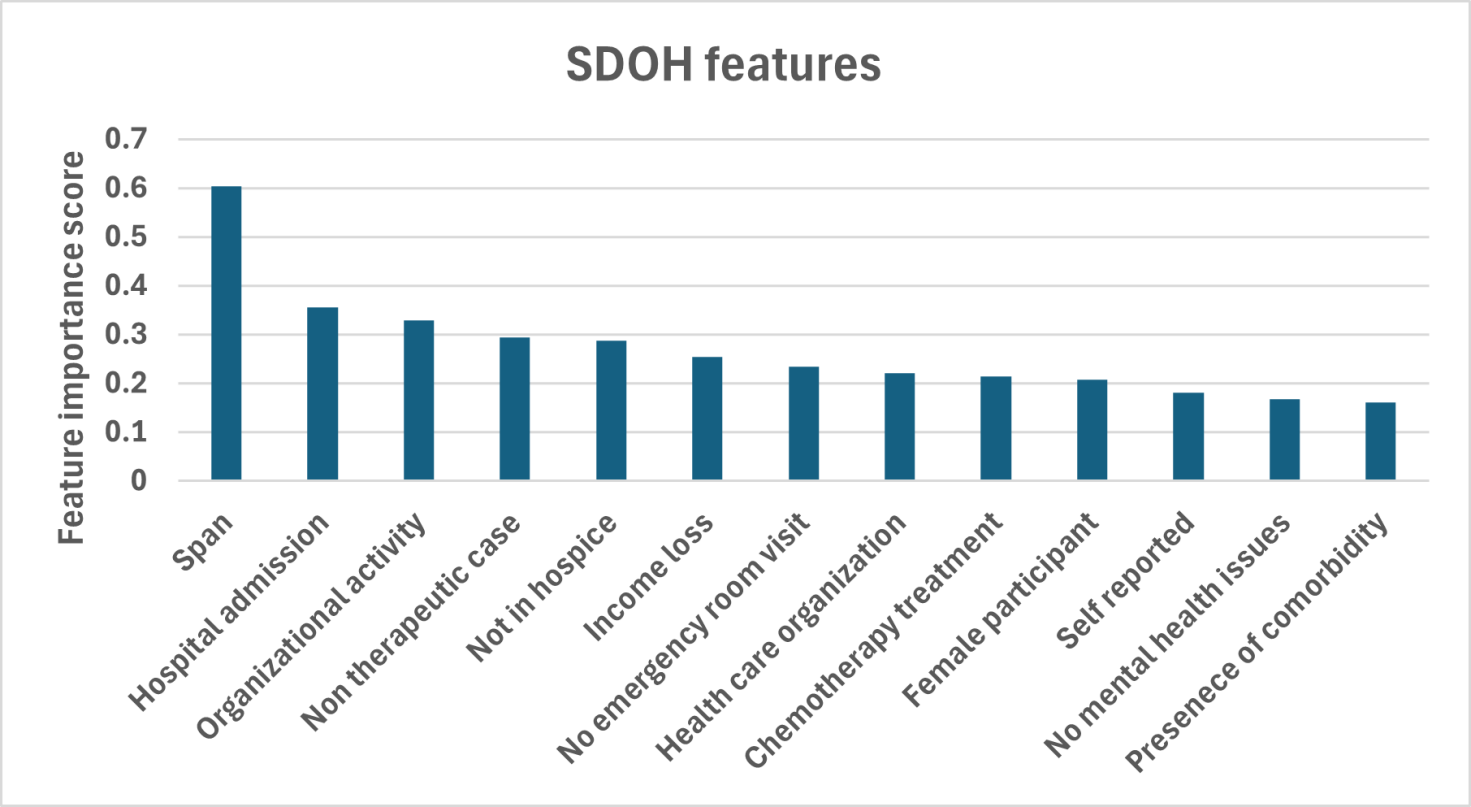
Relative importance of social determinants of health (SDOH) features in predicting crowdfunding campaign success (success ratio threshold=0.7).

Figure 4 shows the top 13 important features (SDOH) in predicting success at a 0.7 success ratio for the gradient boosting algorithm. Permutation importance score is a metric that indicates how much a particular feature contributes to the predictive power of an ML model. On the basis of the chart, the fact that being in the hospital or in unspecified hospice care was highly important highlights the severe medical conditions often associated with crowdfunding campaigns. Policymakers could use this information to identify areas in which the medical system is failing to cover critical care, prompting discussions on improving hospital funding or hospice services. The importance of income loss suggests that financial hardship due to illness is a key driver of crowdfunding success. This implies that patients need better income protection policies or insurance solutions to support individuals who face financial difficulties due to medical conditions. The involvement of health care organizations as a feature suggests that campaigns supported by these organizations might have better access to resources than campaigns organized by individuals. The importance of chemotherapy treatment as a feature suggests that crowdfunding campaigns associated with this treatment might attract more attention and support, possibly because donors recognize the high costs and seriousness of cancer treatment. In addition, female patients in general tend to have more successful campaigns.

**Figure 5.**
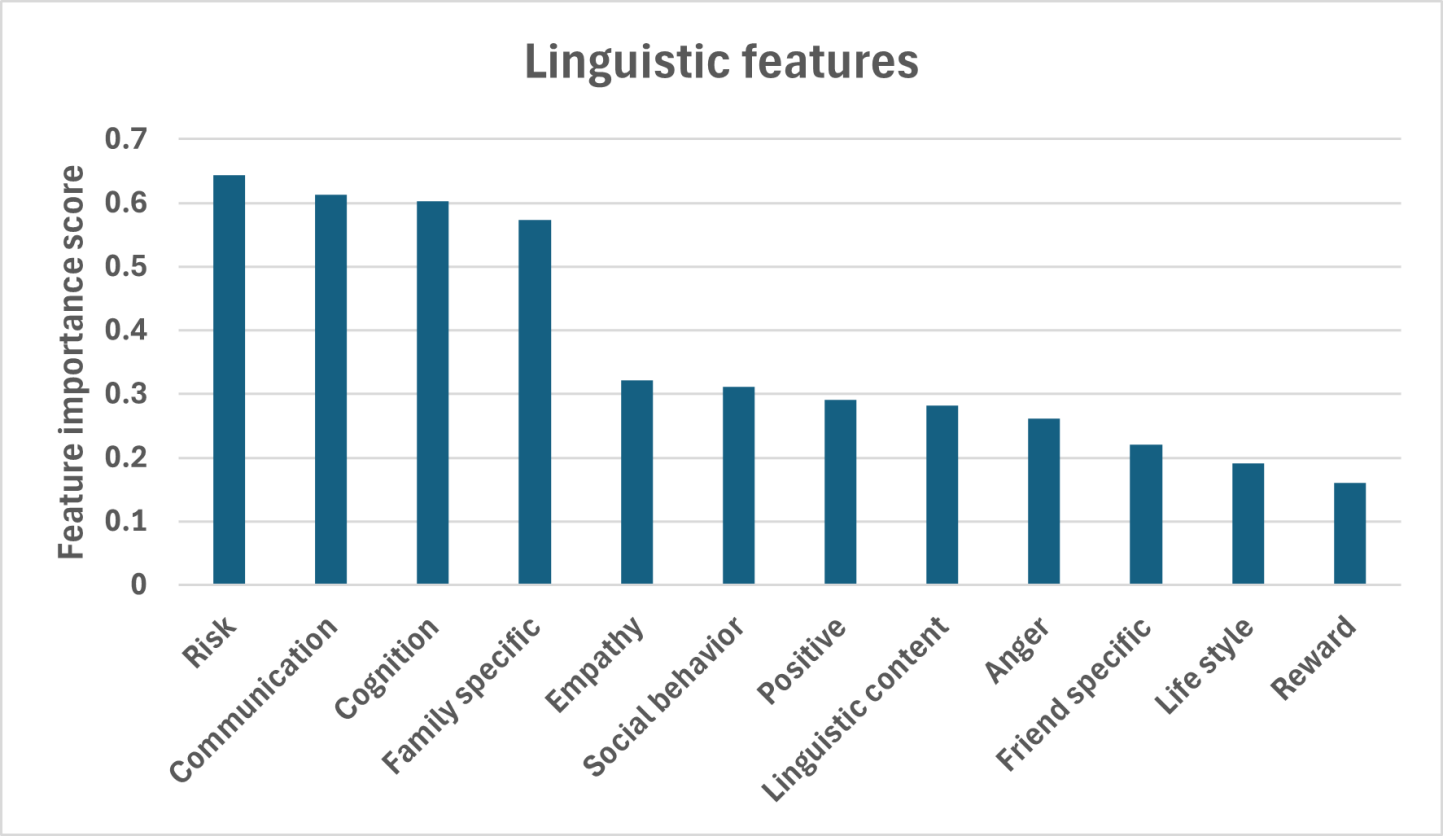
Top linguistic features ranked by permutation importance in predicting campaign success.

Figure 5 shows the permutation importance scores of various linguistic features related to some aspect of behavior or decision-making, possibly in a medical or social context. The features are ranked by their importance in predicting an outcome, with higher scores indicating a greater influence on the model’s performance. The top 4 predictors are risk, clear communication, cognitive style, and family involvement. Empathy and social behaviors are cultivated in professional contexts, and positive messaging should be encouraged across various communication channels. Although less critical, addressing factors such as anger, lifestyle, and reward could further enhance outcomes. These policy recommendations aim to leverage the most influential factors identified by the model to achieve better decision-making and outcomes in the relevant context.

### Performance Evaluation of ML Models

Table 2 and Table 3 present the performance evaluation of 4 ML algorithms—random forest, gradient boosting, logistic regression, and elastic net—at a 0.7 success ratio threshold for SDOH and linguistic factors. The reported performance metrics are accuracy, sensitivity, and specificity across different subsets of top features (ranging from top 3 to top 15). Gradient boosting consistently outperformed the other algorithms in terms of accuracy (consistently 0.786 to 0.788), indicating its superior ability to identify successful crowdfunding campaigns. Random forest offered a balanced performance with good accuracy (0.767 to 0.769) and specificity (0.831 to 0.863), making it a reliable choice for predicting crowdfunding success while capturing nonlinear relationships and interactions. Logistic regression and elastic net exhibited strong specificity (0.997 to 0.999) but poor sensitivity (0.013 to 0.052), which suggests that they may be overly conservative and are not able to predict successful campaigns correctly.

On the basis of the evaluation, when using gradient boosting, selecting the top 3 to 12 features seems optimal. The top 3 features provide a strong balance of high accuracy and sensitivity with fewer variables, making the model simpler and potentially more generalizable. If one aims to maximize model performance with a bit more complexity, one can consider using up to the top 12 features, which slightly enhances specificity without sacrificing other metrics.

**Table 2.** Performance evaluation of machine learning algorithms at a 0.7 success ratio threshold for social determinants of health factors.

Models and metrics		Top 3 features	Top 6 features	Top 9 features	Top 12 features	Top 15 features
Random forest						
	Accuracy	0.77	0.77	0.76	0.78^a^	0.76
	Sensitivity	0.66	0.66	0.65	0.66	0.61
	Specificity	0.83	0.83	0.83	0.84	0.85
Gradient boosting						
	Accuracy	0.79	0.78	0.78	0.79^a^	0.79
	Sensitivity	0.69	0.69	0.69	0.69	0.69
	Specificity	0.84	0.84	0.84	0.84	0.84
Logistic						
	Accuracy	0.64	0.64	0.64	0.64^a^	0.64
	Sensitivity	0.01	0.01	0.01	0.02	0.02
	Specificity	1.00	1.00	1.00	1.00	1.00
Elastic net						
	Accuracy	0.65	0.65	0.65	0.65^a^	0.65
	Sensitivity	0.04	0.05	0.05	0.05	0.05
	Specificity	1.00	1.00	1.00	1.00	1.00

a Italics indicate the highest accuracy values across the 4 algorithms.

**Table 3.** Performance evaluation of machine learning algorithms at a 0.7 success ratio threshold for linguistic factors.

Models and metrics		Top 3 features	Top 6 features	Top 9 features	Top 12 features	Top 15 features
Random forest						
	Accuracy	0.77	0.78	0.78	0.79^a^	0.79
	Sensitivity	0.69	0.69	0.69	0.69	0.67
	Specificity	0.81	0.84	0.84	0.85	0.86
Gradient boosting						
	Accuracy	0.80	0.80	0.80	0.80^a^	0.80
	Sensitivity	0.72	0.73	0.72	0.72	0.72
	Specificity	0.84	0.84	0.84	0.84	0.80
Logistic						
	Accuracy	0.63	0.63	0.63	0.64^a^	0.64
	Sensitivity	0.02	0.03	0.03	0.06	0.06
	Specificity	1.00	1.00	1.00	1.00	1.00
Elastic net						
	Accuracy	0.65	0.65	0.65	0.66^a^	0.66
	Sensitivity	0.08	0.08	0.09	0.11	0.11
	Specificity	1.00	1.00	1.00	1.00	1.00

a Italics indicate the highest accuracy values across the 4 algorithms.

The results indicate that careful feature selection is crucial for optimizing model performance for linguistic features. Gradient boosting and random forest showed that selecting approximately the top 12 features provides a strong balance between sensitivity and specificity, making them well suited for predictive tasks in medical crowdfunding. Logistic regression may require a different approach or fewer features to avoid unnecessary complexity, whereas elastic net’s performance suggests that a moderate feature increase (up to 18-24 features) could be beneficial. Overall, the findings highlight the importance of tailored feature selection strategies to maximize the effectiveness of ML models in this domain.

## Discussion

### Principal Findings

In this study, we conducted an empirical analysis of medical crowdfunding. The analysis of 4984 crowdfunding campaigns revealed key insights into the relationship among goal amounts, campaign success, and donor engagement. Campaigns with higher goals (US $50,000-$100,000) attracted significantly more donors, indicating that ambitious targets may signal substantial projects, thus drawing greater support. Conversely, lower goal amounts (US $799.99-$10,000) were the most prevalent, suggesting a preference for achievable targets among campaigners. Highly successful campaigns had longer durations, averaging over 250 days. We also focused on deriving features using LLMs and feature selection via the random forest algorithm. We identified donors, goal amount, and span of campaign as critical predictors of success, with emotional and thematic features also contributing to campaign success. Among the ML models evaluated, gradient boosting demonstrated superior performance (range 0.786-0.788) and true positive rate (range 0.693-0.696), outperforming random forest, logistic regression, and elastic net. The analysis highlights the critical role of linguistic and SDOH predictors in the success of crowdfunding campaigns. Linguistic features such as clear and effective communication, particularly regarding risk, along with positive messaging, empathy, and social behaviors, significantly influence campaign outcomes. SDOH predictors, including the severity of medical conditions (eg, hospitalization or unspecified hospice care), income loss, and the involvement of health care organizations, are also key drivers of crowdfunding success. These findings suggest a need for improved hospital funding, better income protection policies, and stronger support from health care organizations. In addition, campaigns associated with high-cost treatments such as chemotherapy tend to attract more attention, underscoring the need for targeted support for patients undergoing such treatments.

### Limitations

This study has several limitations. It primarily focused on linguistic, social, demographic, and medical-related features. Other factors, such as donor engagement metrics or campaign visibility on social media platforms, were not evaluated. While cross-validation helps in mitigating overfitting, the generalizability of the model to other types of crowdfunding campaigns beyond the specific dataset used (eg, different medical conditions, geographic regions, or platforms) remains uncertain. The model’s performance may vary when applied to other datasets, limiting its broader applicability. This study relied on the available dataset for training and testing. If the dataset is not representative of all cancer crowdfunding campaigns (eg, biased toward certain demographics, treatment types, or socioeconomic groups), the model may not perform well in more diverse or less represented scenarios. Ultimately, the default parameters we chose for random forest feature selection due to a superior performance than that of the grid search method may not be applicable or effective for different datasets.

### Comparison With Previous Studies

The findings that communication regarding risk, social behaviors, and empathy play a crucial role in influencing donor behavior have been documented in a previous study [32], where it was found that emotionally compelling narratives significantly improve crowdfunding outcomes. Research highlighting the financial burden [33] of cancer care provides further support for income loss as a significant predictor. The importance of factors such as being in a hospital or receiving chemotherapy treatment is supported by studies showing that campaigns related to severe or high-cost treatments, such as advanced-stage cancers, often receive more attention and donations [34]. Our study also sheds light on new insights on medical crowdfunding. The analysis introduces the importance of specific linguistic features, such as the use of positive language and social behavior indicators, which have not been as thoroughly explored in previous studies. The involvement of health care organizations as a significant predictor suggests that campaigns backed by institutions might have better access to resources and are perceived as more credible.

### Conclusions

The reliance on crowdfunding for conditions associated with high-importance features such as hospital stays and income loss highlights potential gaps in health care funding. Policymakers need to assess where these gaps are and consider reforms or additional support mechanisms to reduce the need for crowdfunding in critical areas. The variability in the importance of different features may also point to inequities in access to health care and financial support. Policymakers can better target support to vulnerable populations such as those with comorbidities. A crucial policy objective is ensuring that all individuals, regardless of their medical or financial situation, have equal access to medical care without crowdfunding.

## Abbreviations

API: application programming interface
LLM: large language model
ML: machine learning
SDOH: social determinants of health
